# Development of latent fingerprints on non-porous surfaces recovered from fresh and sea water

**DOI:** 10.1186/s41935-017-0008-8

**Published:** 2017-07-18

**Authors:** Somaya Madkour, Abeer sheta, Fatma Badr El Dine, Yasser Elwakeel, Nermine AbdAllah

**Affiliations:** 1 Department of Forensic Medicine and Clinical Toxicology, Faculty of Medicine, University of Alexandria, Azarita, Alexandria, Egypt; 2 Department of Physical Oceanography, Faculty of Science, University of Alexandria, Elanfoushi, Alexandria, Egypt

**Keywords:** Fingerprint, Cyanoacrylate, Sea water, Fresh water, Non-porous

## Abstract

**Background:**

Criminal offenders have a fundamental goal not to leave any traces at the crime scene. Some may suppose that items recovered underwater will have no forensic value, therefore, they try to destroy the traces by throwing items in water. These traces are subjected to the destructive environmental effects. This can represent a challenge for forensic experts investigating fingerprints.

**Methods:**

The present study was conducted to determine the optimal method for latent fingerprints development on dry non-porous surfaces submerged in aquatic environments at different time interval. The quality of the developed fingerprints depending on the used method was assessed. In addition, two factors were analyzed in this study; the effects of the nature of aquatic environment and the length of submerged time. Therefore, latent fingerprints were deposited on metallic, plastic and glass objects and submerged in fresh and sea water for 1, 2, and 10 days. After recovery, the items were processed by black powder, small particle reagent and cyanoacrylate fuming and the prints were examined. Each print was evaluated according to fingerprint quality assessment scale.

**Results:**

Cyanoacrylate developed latent prints found to have the highest mean visibility score after submersion in fresh and sea water for 1, 2 and 10 days. Mean visibility score of prints developed showed significant decline after 10 days of submersion. Prints submerged in fresh water showed significantly higher mean visibility score than those submerged in sea water using various methods of development and in all time intervals.

**Conclusion:**

The study demonstrated that it is possible to recover latent prints submerged in water on different studied dry non porous surfaces with the best visualization method using cyanoacrylate either in fresh or sea water. The duration of submersion affects the quality of fingerprints developed; the longer the duration, the worse the quality is. In addition, this study has revealed that the exposure to high salinity i.e. sea water has more damaging influence on the quality of detected fingerprints.

It is concluded that any piece of evidence recovered from underwater should be tested for prints, no matter the amount of time spent beneath the surface.

## Background

In spite of the developments made in DNA profiling, fingerprints are still considered as the most widely established forms of forensic evidence used by law to certainly identify an individual (Kapoor et al. 2015). Criminal offenders have a fundamental goal not to leave any traces at the crime scene. Some may believe that items recovered underwater will have no forensic value; therefore, they try to destroy these traces by throwing items in water (Trapecar 2012a). Therefore, it has been the concern of forensic authorities to examine evidences recovered from different aquatic environments. Criminals and law enforcement have been amazed by the physical evidence that remains preserved despite the duration of submersion (Popov et al. 2017).

Natural fingerprint residue is composed of a mixture of numerous substances; 99% water and the remaining part consist of small amount of organic and inorganic materials (Girod et al. 2012). Non-porous surfaces do not absorb moisture. Latent prints on these substrates are more susceptible to damage as the fingerprint residue existing on the outermost surface is more subjected to environmental factors (Yamashita and French 2011; Almog et al. 2004).

Several studies demonstrated various factors that may influence the quality of developed latent prints in water including; individual variation of latent fingerprint composition, the nature of surface, time elapsed since deposition, environmental factors; such as air circulation, dust, humidity, light exposure, precipitation, temperature, ultraviolet rays and the enhancement techniques. The composition of fingerprints also changes over time that may affect the efficiency of development techniques (Girod et al. 2012; Archer et al. 2005; Croxton et al. 2010).

Trapecar M, Jasuja et al. and Castello et al. assessed the effect of fresh water on the quality of developed latent prints using various methods of development, they found that latent prints are still could be recovered from submerged substrates and that fresh water do not have major destructive effect (Trapecar 2012b; Jasuja et al. 2015; Castello´ et al. 2013).

Some of the optimal techniques that was proved to be effective in latent prints developments on non-porous surfaces are: Black powder, Small Particle Reagent and Cyanoacrylate fuming, Vacuum metal deposition (Polimeni et al. 2004; Rohatgi and Kapoor 2016; Olenik 1984).

### Black powder

Black powder is one of the first and most common methods of latent print detection. It is composed of a variety of carbon-based powders with a binder added for stability. Finely divided particles physically adhere to water and oily residues of fingerprints (Lee and Gaensslen 2001).

### Small particle reagent (SPR)

SPR is a suspension of molybdenum disulfide (MoS_2_) in a detergent solution (Kapoor et al. 2015).

SPR works by physical adhesion to fatty residues forming a gray deposit. SPR is available commercially in a pre-mixed liquid form. Jasuja et al., Rohatgi et al. concluded that SPR is one of the most suitable methods of latent print development on non-porous surfaces especially on wet surfaces (Jasuja et al. 2015; Rohatgi and Kapoor 2016); although SPR seems to perform equally well on dry and wet surfaces (Cuce et al. 2004).

### Cyanoacrylate (CA)

Since the late 1970s, Cyanoacrylate fuming (super glue) continues to be adaptable, effective and popular development technique on almost all non-porous surfaces and some porous surfaces. Paine et al. demonstrated how Cyanoacrylate vapor is selectively attracted to fingerprint residues, where it polymerizes on the fingerprint ridges to form a hard, white polymer known as poly- ethylcyanoacrylate (PECA). The study also clarified the importance of humidity and its effect on cyanoacrylate enhancement being a primary initiator of polymerization (Paine et al. 2011). Fully developed CA prints are a white three-dimensional matrix, often visible to the unaided eye, and can be further enhanced with a variety of techniques. This method should to be done in an enclosed space to enclose the fumes and because oxygen is considered a terminating agent for the polymerization process. Fuming with cyanoacrylate can be achieved by several means, ranging from inexpensive home-made chambers to large expensive commercial units (Dadmun 2009; Wargacki et al. 2008).

To the best of our knowledge; none of the previous studies in the field compared the effect of fresh and sea water on latent print development. Therefore the aim of the present study was to make such comparison together with detecting the best method of visualization (black powder, SPR and CA) with variable time intervals.

## Methods

### Material used

- Non-porous surfaces used:
  - Glass sheets (approx. 20 × 10 cm)
  - Compact discs (shiny surface)
  - Knife blades (stainless steel)
- Methods used for prints development:
  - Black powder: BPP2018 Silver/Black “Hi-Fi” Latent Print Powder, 8 oz. (237 mL), Sirchie Co. The brush used: 120LS Squirrel Hair brush with dimensions; Handle length: 4 5/8” (11.75 cm), Brush Length: 1 3/4” (4.45 cm).
  - Small particle reagent (SPR): 100 Dark SPR w/spray head, 500 ml, Sirchie Co.
  - Cyanoacrylate: CA102 OMEGA-PRINT™ Cyanoacrylate Fuming Compound, 20 g, Sirchie Co.
- Aquarium tank formed of glass (1 m x 1 m x 0.7 m), with portable battery air pump and fan for aquatic simulation.
- Home-made Cyanoacrylate chamber: plastic box 70 x 40 x 40 cm (lined from inside by aluminum foil) with electric single cooking plate and cup of warm water (humidity is an important factor).
- Alcohol, water spray, permanent marker, gloves and magnifying glass were used in development and examination.

### Methods

The current study was conducted in both sea and fresh water.

Eighteen glass plates, 18 plastic surfaces (CDs) and 18 knife blades were used for each of the sea and fresh water experiments.

#### Simulation/Modelling of natural aquatic environment

The study was conducted in winter season (air temperature 12–14 °C, relative humidity 88.4%). The aquarium tank is filled with the water type of study (water temperature 18 °C). Its bottom (5 cm) was filled with sea-bed sand and lake-bed mud when applying sea and fresh water respectively.

The portable battery air pump is turned on daily (1 h) for water oxygenation, and a fan directed tangentially at the top of water to simulate the laminar flow of water made by wind.

Sea water was obtained from Elshatby region, Mediterranean Sea (latitude 31°12'40.60"N, longitude 29°54'45.99"E) while fresh water was obtained from Lake Racta- branch from Elmahmodia Lake (latitude 31°16'23.35"N, longitude 30° 5'4.61"E).

#### Fingerprint deposition

Each non porous surface was cleaned by alcohol swabs to make sure no unintentional prints were deposited.

Informed consent from five fingerprint donors was taken. The fingerprint donors were informed not to wash their hands before the experiment. They were asked to rub their fingertip against the forehead and around the nose (groomed/ sebum rich fingerprint), then press their fingers in rolling motion against the surface.

Fingers of donors were applied to the surface by the researcher to ensure that fingertip area sampled, time of contact and pressure were as consistent as possible between donors.

Five groomed fingerprints were deposited in depletion series on each surface. The intention was to initially deposit good quality fingerprints onto the substrate and, where possible, oblique lighting was used to confirm that the quality and clarity of those recently deposited fingerprints were identifiable. The deposited prints were labeled using permanent marker.

**Fig. 1:**
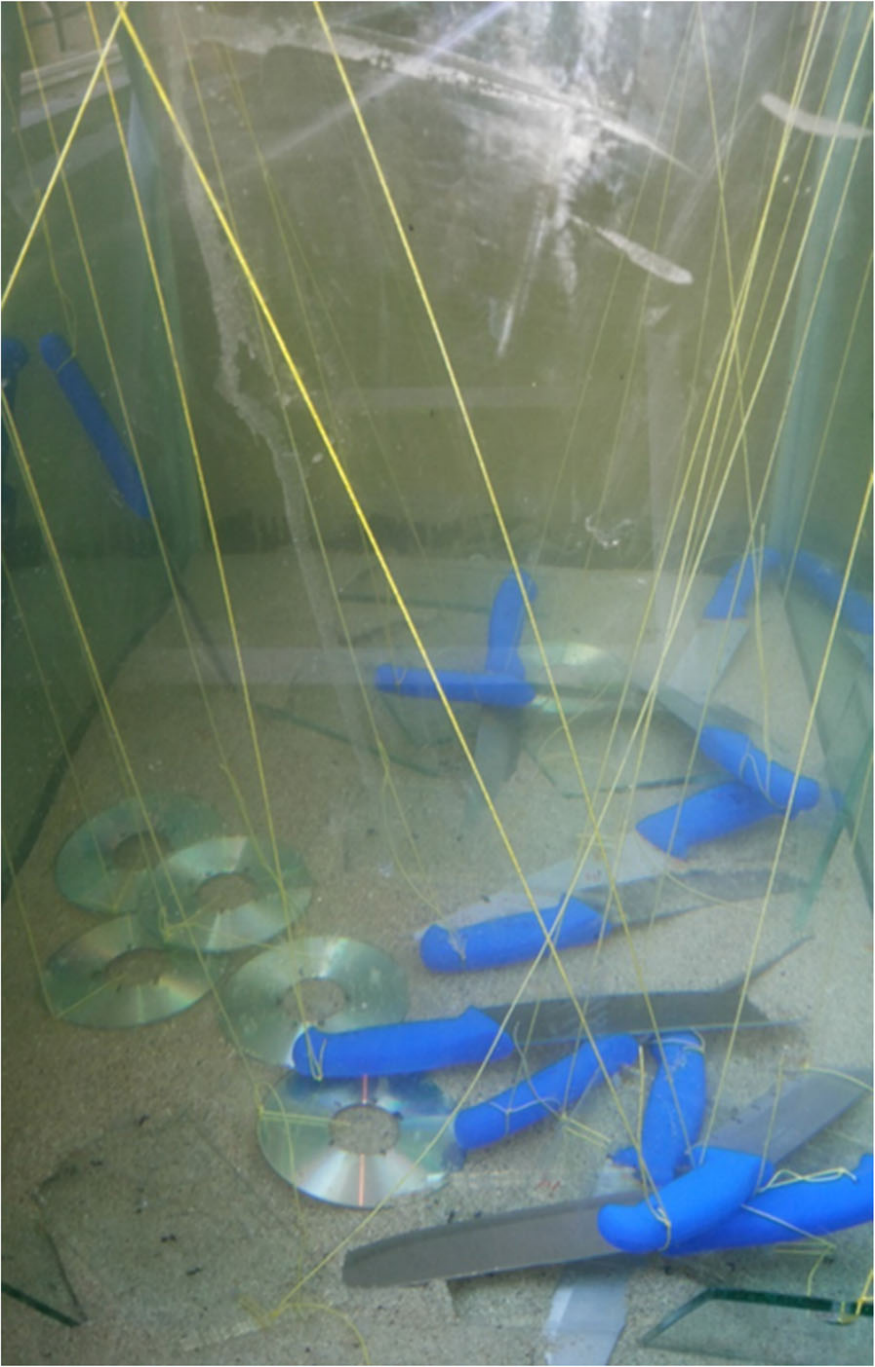
non porous surfaces were placed in aquarium tank filled with type of water of study after fingerprints deposition

Eighteen surfaces (of each material) were placed one hour later in the tank filled with water of study (Fig. 1). Eight surfaces were recovered from water after each interval; 1, 2 and 10 days. They are then left in room air to be dried. Each two surfaces, with ten finger marks, were examined with enhancement techniques of black powder, SPR and CA.

#### Methods of visualization

The surfaces were left in air for two hours to dry then the following methods were used:

##### Dusting technique

**Fig. 2:**
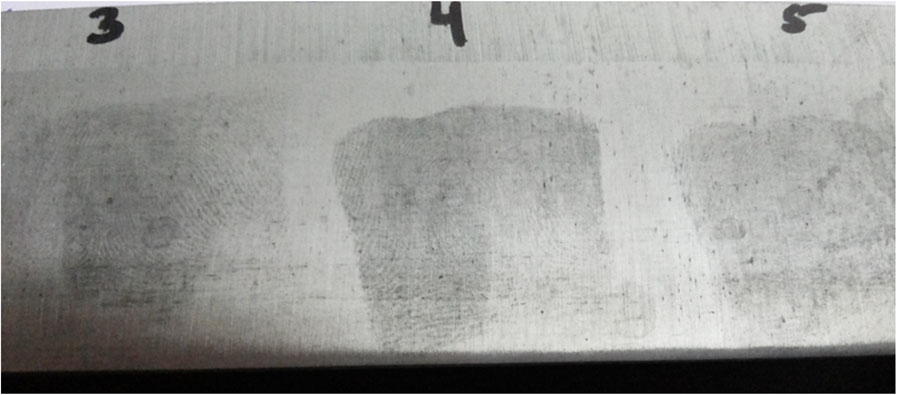
Developed latent fingerprints using *black powder* on knife blade after submersion in fresh water for 2 days. (Score 3)

**Fig. 3:**
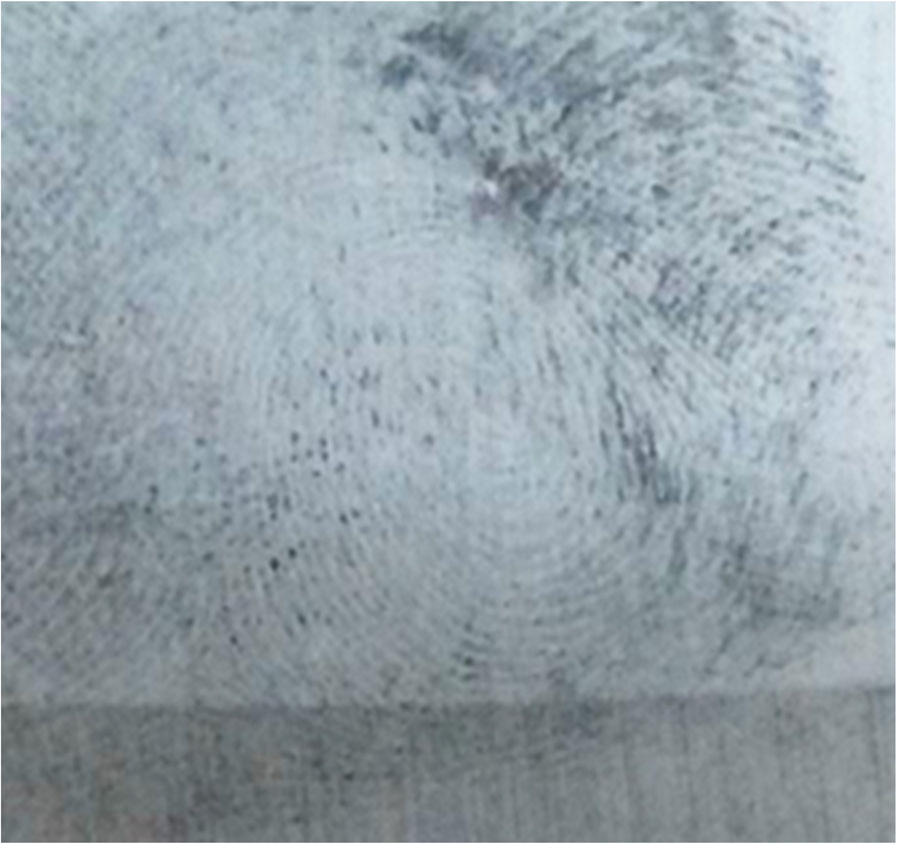
Developed fingerprint using *black powder* on glass surface after submersion in sea water for 1 day. (Score 3)

Little amount of the black powder was sprinkled on the non-porous surface and the excess was removed using the squirrel hair brush with special care to leave the fingerprints intact (Figs. 2 and 3).

##### Small Particle Reagent (SPR) technique

**Fig. 4:**
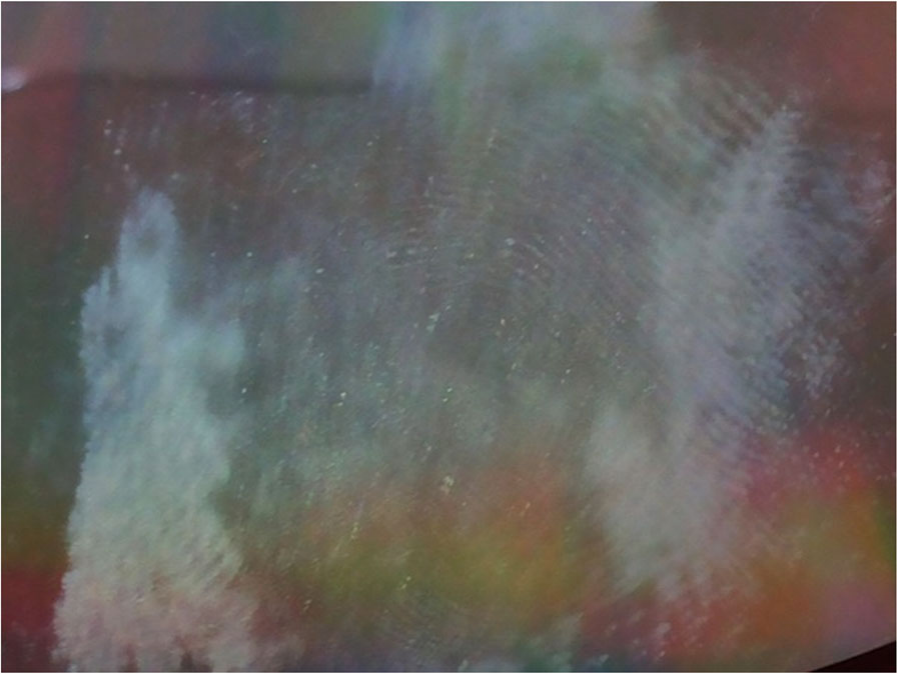
Developed fingerprints using SPR on plastic surface (CD) after submersion in fresh water for 2 days (Score 2)

SPR bottle was shaken vigorously before spraying from the top of the non-porous surface downwards to prevent displacement of latent print. The formulation is left for one minute to react with print residue then the excess SPR was washed using water spray (Fig. 4).

##### Cyanoacrylate (CA) fuming technique

**Fig. 5:**
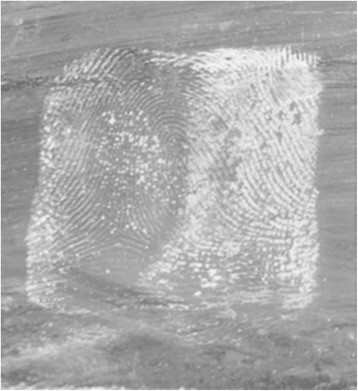
Developed fingerprint using cyanoacrylate on glass surface after submersion in fresh water for 1 day (Score 5)

Four to five drops of CA were placed in aluminum cup on the electric cooking plate. The glass, plastic and metal surfaces were placed in the closed chamber. The electric plate was put on for 5 min which was found to be appropriate time for developing the latent prints. The procedure is repeated for every experiment under the same conditions, temperature and degree of humidity (Fig. 5).

Gloves were used during handling of objects in the whole previous steps except at time of donor fingerprint deposition to avoid unwanted prints.

#### Fingerprints examination

The developed latent prints were examined using magnifying glass and photographed. All print marks were examined, assessed and scored according to fingerprint quality assessment scale (Castello´ et al. 2013; Soltyszewski et al. 2007; Devlin 2011):

##### Score 5- Very good visibility

Clearly defined friction ridges across entire print. Classifiable as one of the three basic fingerprint patterns (arch, loop, or whorl). Core (center point) and minutiae (individual features, e.g. bifurcation, ending ridge) are visible.

##### Score 4- Good visibility

Clearly defined friction ridges are visible across majority of print. Classifiable as one of the three basic fingerprint patterns (arch, loop, or whorl).

##### Score 3- Poor visibility

Friction ridges are only visible on portion of print. The print cannot be classified into one of the three basic fingerprint patterns. Prints may be smudged.

##### Score 2- Bad visibility

No friction ridges are clearly defined. Print is almost completely smudged or obscured and cannot be classified into one of the three basic fingerprint patterns.

##### Score 1- Blur/No print

No print is visible or only the outline of print is visible.

## Statistical analysis

Data were fed to the computer and analyzed using IBM SPSS software package version 20.0.(2) Qualitative data were described using number and percent. Quantitative data were described using range (minimum and maximum) mean, standard deviation. For normally distributed data, comparison between the two studied groups were done using independent t-test while F-test (ANOVA) was used and Post Hoc test (LSD) to compare between the three studied groups. Significance of the obtained results was judged at the 5% level (Kotz et al. 2006; Kirkpatrick and Feeney 2003).

## Results

### Sea water

#### Black powder

The quality of 40% of the developed marks on glass surface was good either in first or second day. Yet, No prints were detected on 10th day. Regarding metal or plastic surface, no marks with good visibility were detected in day 1, 2 or 10 (Table 1).

**Table 1:** Fingerprints development scores using **black powder technique** on glass, metal and plastic surfaces submerged in **sea water** at 1, 2 and 10 days’ intervals according to fingerprints quality assessment scale

Black powder	Time (days)	Number of deposited marks	Scores									
			5 (very good)		4 (good)		3 (poor)		2 (bad)		1 (blur/no)	
			N	%	n	%	N	%	n	%	n	%
Glass	1	10	0	0%	4	40%	6	60%	0	0%	0	0%
	2	10	0	0%	4	40%	5	50%	1	10%	0	0%
	10	10	0	0%	0	0%	0	0%	0	0%	10	100%
Metal	1	10	0	0%	0	0%	3	30%	6	60%	1	10%
	2	10	0	0%	0	0%	3	30%	4	40%	3	30%
	10	10	0	0%	0	0%	2	20%	1	10%	7	70%
Plastic	1	10	0	0%	0	0%	2	20%	3	30%	5	50%
	2	10	0	0%	0	0%	2	20%	2	20%	6	60%
	10	10	0	0%	0	0%	2	20%	2	20%	6	60%

#### SPR

The quality of the developed fingerprints on glass surfaces after 1 day revealed that half of them were of poor visibility.

On metal surface, the quality of 40% of the developed marks were with poor visibility and most of the prints were blur and absent after 1 day exposure (Table 2).

**Table 2:** Fingerprints development scores using **SPR technique** on glass, metal and plastic surfaces submerged in **sea water** at 1, 2 and 10 day’s intervals according to fingerprints quality assessment scale

SPR	Time (days)	Number of deposited marks	Scores									
			5 (very good)		4 (good)		3 (poor)		2 (bad)		1 (blur/no)	
			N	%	n	%	n	%	n	%	n	%
Glass	1	10	0	0%	0	0%	5	50%	5	50%	0	0%
	2	10	0	0%	0	0%	3	30%	1	10%	6	60%
	10	10	0	0%	0	0%	0	0%	0	0%	10	100%
Metal	1	10	0	0%	1	10%	4	40%	5	50%	0	0%
	2	10	0	0%	0	0%	2	20%	3	30%	5	50%
	10	10	0	0%	0	0%	2	20%	4	40%	4	40%
Plastic	1	10	0	0%	2	20%	2	20%	3	30%	3	30%
	2	10	0	0%	0	0%	0	0%	2	20%	8	80%
	10	10	0	0%	0	0%	1	10%	2	20%	7	70%

#### CA fuming

Sixty percent of the prints were developed on glass surfaces with good visibility after 1 day of submersion. On the second day, only half of the developed prints were of good visibility.

Regarding the metal surface, 50% of the fingerprints were obtained with good visibility by the first day. On the other hand; after 10 days of submersion, most of the prints were invisible (Table 3).

**Table 3:** Fingerprints development scores using **CA technique** on glass, metal and plastic surfaces submerged in **sea water** at 1, 2 and 10days’ intervals according to fingerprints quality assessment scale

CA	Time (days)	Number of deposited marks	Scores									
			5 (very good)		4 (good)		3 (poor)		2 (bad)		1 (blur/no)	
			n	%	n	%	n	%	n	%	n	%
Glass	1	10	2	20%	6	60%	2	20%	0	0%	0	0%
	2	10	0	0%	5	50%	5	50%	0	0%	0	0%
	10	10	0	0%	0	0%	4	40%	6	60%	0	0%
Metal	1	10	3	30%	5	50%	2	20%	0	0%	0	0%
	2	10	3	30%	6	60%	3	10%	0	0%	0	0%
	10	10	0	0%	0	0%	0	0%	3	30%	7	70%
Plastic	1	10	0	0%	6	60%	4	40%	0	0%	0	0%
	2	10	0	0%	3	30%	2	20%	0	0%	5	50%
	10	10	0	0%	2	20%	5	50%	3	30%	0	0%

**Fig. 6:**
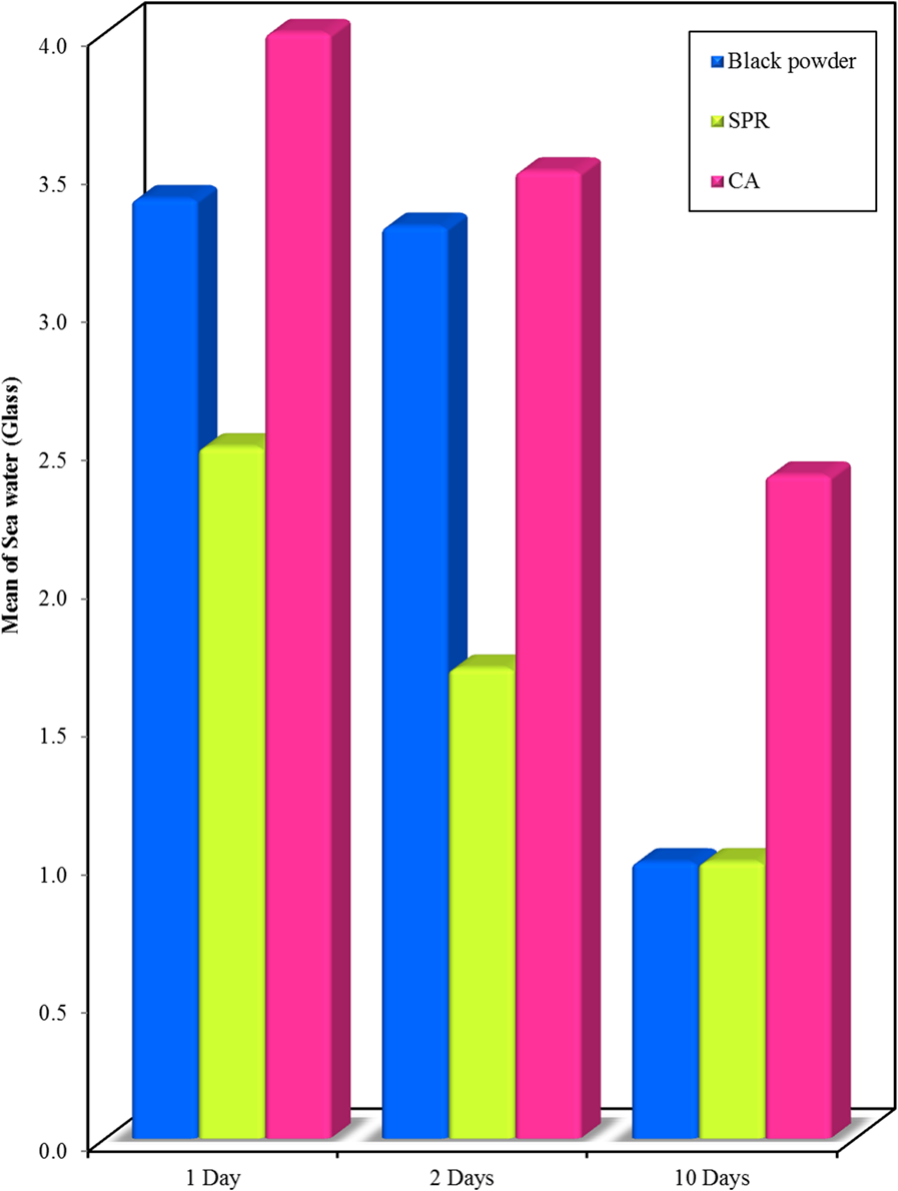
Bar graph - Comparison between the Black powder, SPR and CA development techniques at different time intervals (1, 2, and 10 days) on **glass surface** recovered from **sea water**. Showing highest mean visibility score with CA

**Fig. 7:**
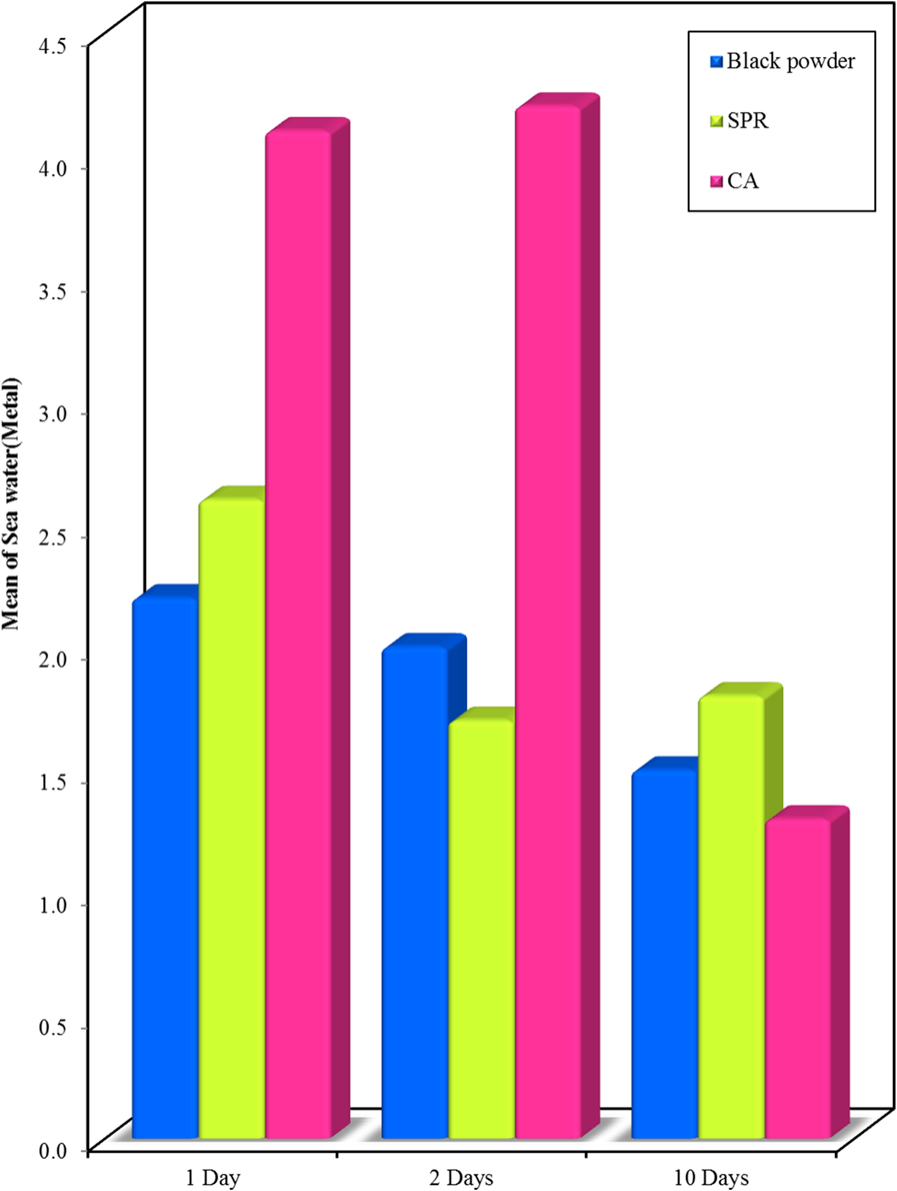
Bar graph - Comparison between the Black powder, SPR and CA development techniques at different time intervals (1, 2, and 10 days) on **metal surface** recovered from **sea water**. It shows higher mean visibility score after using CA in 1 and 2 day’s intervals

**Fig. 8:**
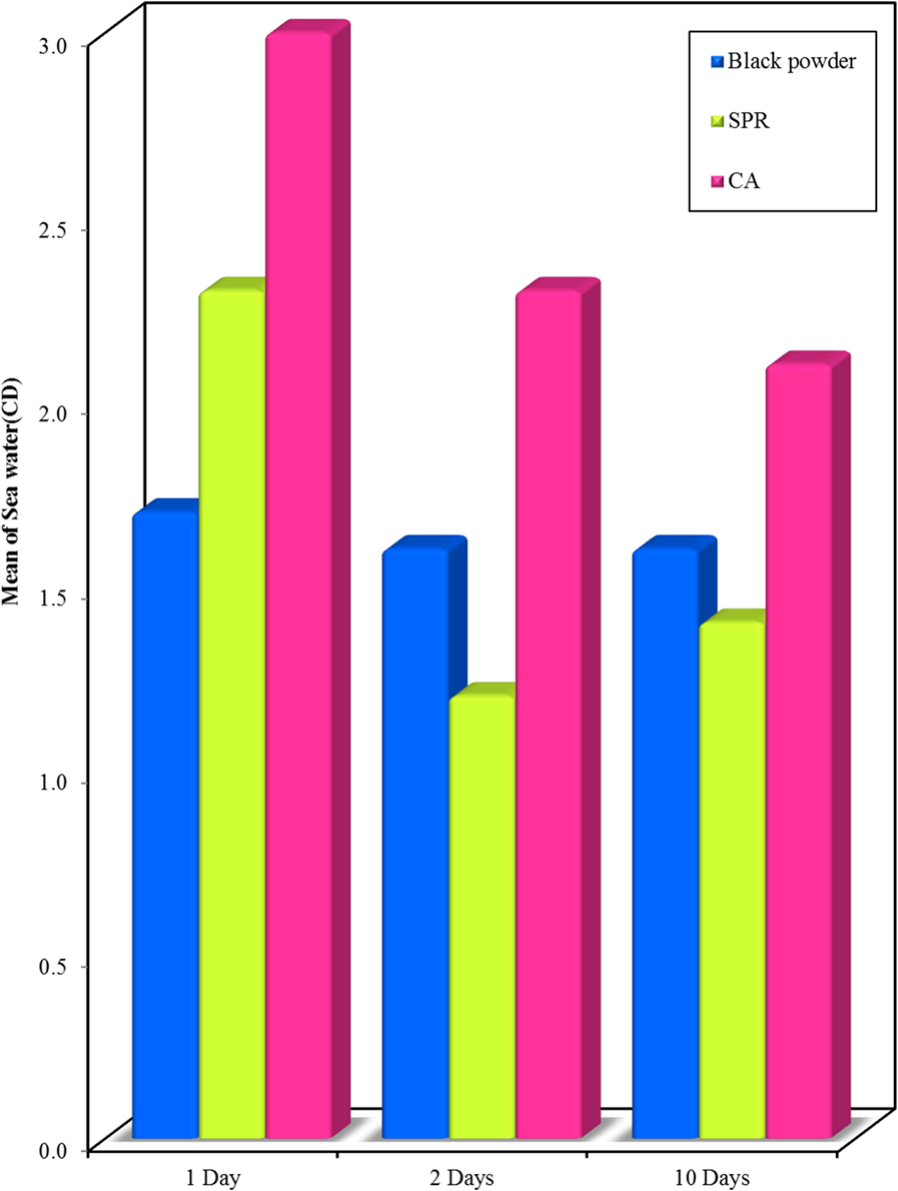
Bar graph - Comparison between the Black powder, SPR and CA development techniques at different time intervals (1, 2, and 10 days) on **plastic surface** (CD) recovered from **sea water**. It shows higher mean visibility score of prints developed using CA

Significant differences (*P* < 0.001) were observed among all methods on different materials, with the highest mean score obtained upon using CA technique (Figs. 6, 7 and 8).

### Fresh water

#### Black powder

Half of the developed prints on glass surface were of good visibility (50%). On the metal surface, the first day of exposure showed 70% of good and very good visibility marks as revealed from Table 4.

**Table 4:** Fingerprints development scores using **black powder technique** on glass, metal and plastic surfaces submerged in **fresh water** at 1, 2and 10 days’ intervals according to fingerprints quality assessment scale

Black powder	Time (days)	Number of deposited marks	Scores									
			5 (very good)		4 (good)		3 (poor)		2 (bad)		1 (blur/no)	
			n	%	N	%	n	%	n	%	N	%
Glass	1	10	4	40%	5	50%	1	10%	0	0%	0	0%
	2	10	2	20%	7	70%	1	10%	0	0%	0	0%
	10	10	0	0	0	0	5	50%	1	10%	4	40%
Metal	1	10	1	10%	6	60%	3	30%	0	0%	0	0%
	2	10	0	0%	2	20%	6	60%	2	20%	0	0%
	10	10	0	0%	0	0%	2	20%	5	50%	3	30%
Plastic	1	10	0	0	4	40%	4	40%	2	20%	0	0%
	2	10	0	0	0	0%	5	50%	5	50%	0	0%
	10	10	0	0	0	0%	0	0%	2	20%	8	80%

Table 4 also shows that; on plastic surface, 40% of prints were of good visibility on the first day.

#### SPR

After 1 day of submersion; the quality of the developed fingerprints on glass surfaces was of good visibility in 80% while on metal surfaces 60% was of poor visibility.

Regarding the plastic surface, only 20% of the marks were of good visibility on the first day or second day of exposure (Table 5).

**Table 5:** Fingerprints development scores using **SPR technique** on glass, metal and plastic surfaces submerged in **fresh water** at 1, 2 and 10 days’ intervals according to fingerprints quality assessment scale

SPR	Time (day)	Number of deposited marks	Scores									
			5 (very good)		4 (good)		3 (poor)		2 (bad)		1 (blur/no)	
			n	%	N	%	n	%	n	%	n	%
Glass	1	10	0	0%	8	80%	2	20%	0	0%	0	0%
	2	10	0	0%	6	60%	3	30%	1	10%	0	0%
	10	10	0	0%	0	0%	1	10%	6	60%	3	30%
Metal	1	10	0	0%	3	30%	6	60%	1	10%	0	0%
	2	10	0	0%	2	20%	5	50%	3	30%	0	0%
	10	10	0	0%	3	30%	3	30%	4	40%	0	0%
Plastic	1	10	0	0%	2	20%	3	30%	5	50%	0	0%
	2	10	0	0%	2	20%	4	40%	4	40%	0	0%
	10	10	0	0%	0	0%	6	60%	1	10%	3	30%

#### CA fuming

One day after submersion in fresh water, 80% of prints were developed on glass surfaces with very good visibility.

Regarding the metal surfaces, all the developed fingerprints showed good and very good visibility after 1 or 2 days of exposure to fresh water. On plastic material, 60% of fingerprints were of good visibility after 1 day of submersion (Table 6).

**Table 6:** Fingerprints development scores using **CA technique** on glass, metal and plastic surfaces submerged in **fresh water** at 1, 2 and 10days’ intervals according to fingerprints quality assessment scale

CA	Time (day)	Number of deposited marks	Scores									
			5 (very good)		4 (good)		3 (poor)		2 (bad)		1 (blur/no)	
			N	%	N	%	n	%	n	%	N	%
Glass	1	10	8	80%	2	20%	0	0%	0	0%	0	0%
	2	10	4	40%	5	50%	1	10%	0	0%	0	0%
	10	10	0	0%	3	30%	5	50%	2	20%	0	0%
Metal	1	10	6	60%	4	40%	0	0%	0	0%	0	0%
	2	10	4	40%	6	60%	0	0%	0	0%	0	0%
	10	10	0	0%	6	60%	3	30%	1	10%	0	0%
Plastic	1	10	0	0%	6	60%	4	40%	0	0%	0	0%
	2	10	0	0%	2	20%	3	30%	5	50%	0	0%
	10	10	0	0%	2	20%	5	50%	3	30%	0	0%

**Fig. 9:**
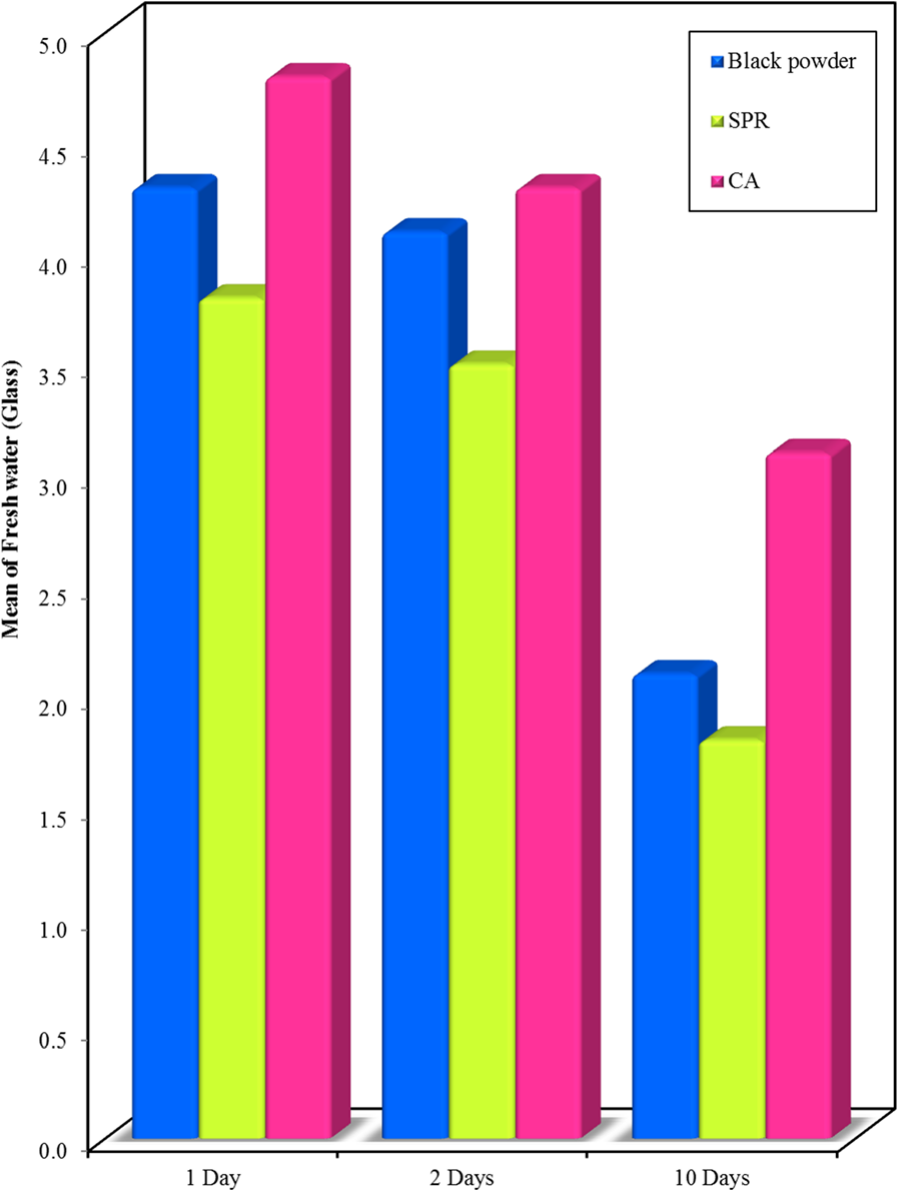
Bar graph - Comparison between the Black powder, SPR and CA development techniques at different time intervals (1, 2, and 10 days) on **glass surface** recovered from **freshwater**. It shows that highest mean visibility score of prints developed using CA

**Fig. 10:**
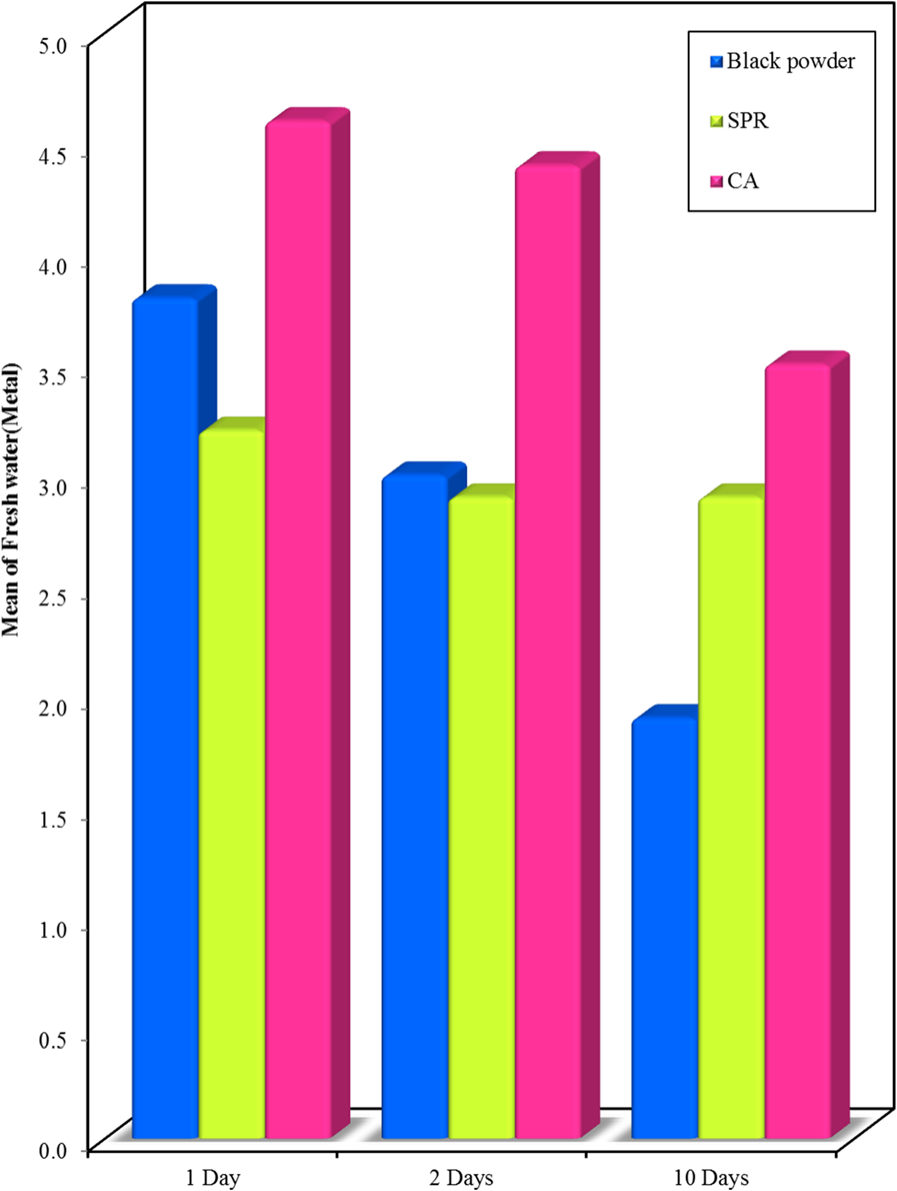
Bar graph - Comparison between the Black powder, SPR and CA development techniques at different time intervals on **metal surface** recovered from **freshwater**. It shows that highest mean visibility score of prints developed using CA

**Fig. 11:**
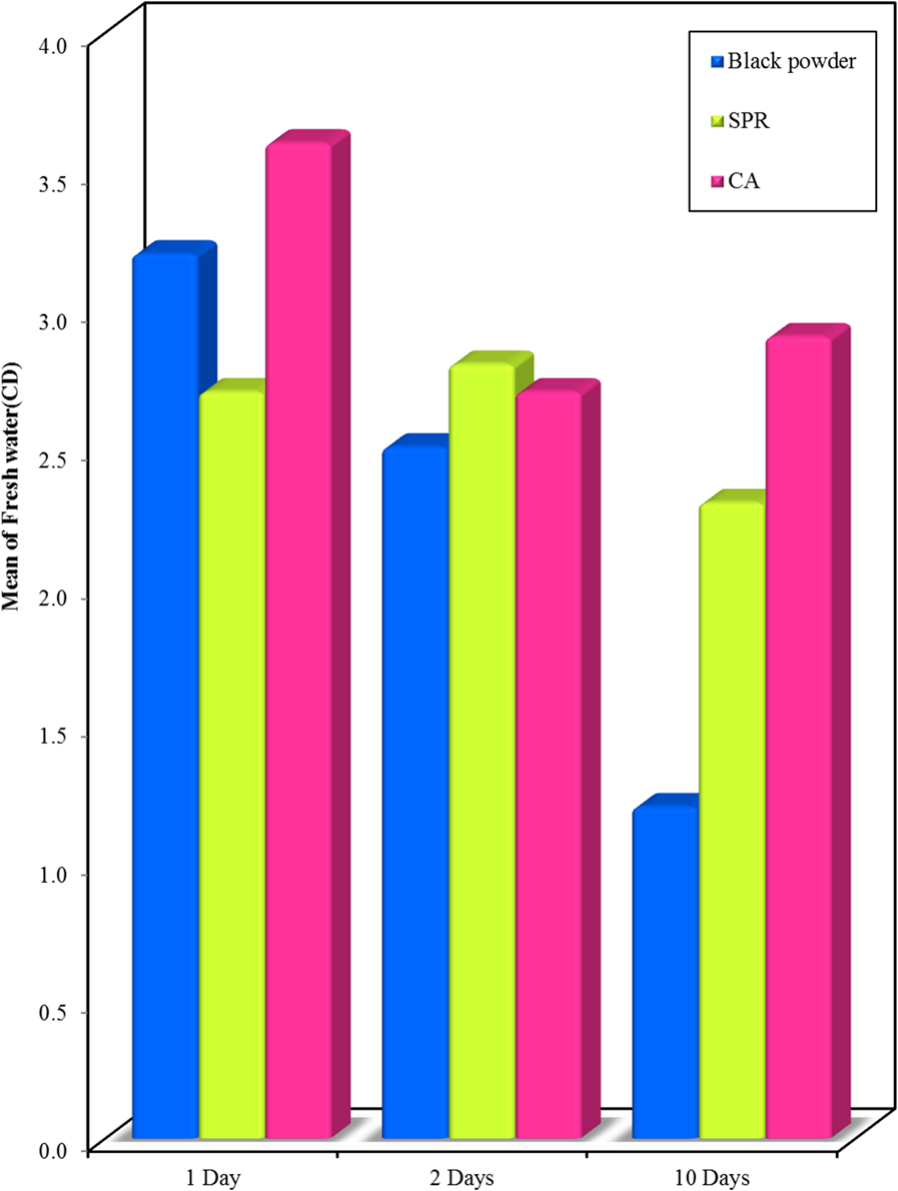
Bar graph - Comparison between the Black powder, SPR and CA development techniques at different time intervals on **plastic surface** recovered from **freshwater**. It shows that highest mean visibility score of prints developed using CA

On comparing the three studied techniques used for fingerprints development after submergence in fresh water, a significant difference in the majority of examined prints was noticed with the highest score when using CA (Figs. 9, 10 and 11).

On the basis of the results; CA was found to be the best technique used for development of latent fingerprints on different dried studied materials recovered from sea or fresh water, sea water is considered more destructive to fingerprints than fresh water (Table 7). Also it was found that the quality of fingerprints developed was affected by the duration of submersion.

**Table 7:** Comparison between the mean fingerprints development **scores** of the studied groups according to **aquatic environment** and the used **techniques** for development of latent fingerprints from different **surfaces** at **different time intervals**

		Sea water (*n* = 10)	Fresh water (*n* = 10)	T	*p*
Glass	Black powder				
	Day 1	3.40 ± 0.52	4.30 ± 0.67	3.349^*^	0.004^*^
	Day 2	3.30 ± 0.67	4.10 ± 0.57	2.869^*^	0.010^*^
	Day 10	1.0 ± 0.0	2.10 ± 0.99	3.498^*^	0.007^*^
	SPR				
	Day 1	2.50 ± 0.53	3.80 ± 0.42	6.091^*^	<0.001^*^
	Day 2	1.70 ± 0.95	3.50 ± 0.71	4.811^*^	<0.001^*^
	Day 10	1.0 ± 0.0	1.80 ± 0.63	4.000^*^	0.003^*^
	CA				
	Day 1	4.0 ± 0.67	4.80 ± 0.42	3.207^*^	0.008^*^
	Day 2	3.50 ± 0.53	4.30 ± 0.67	2.954^*^	0.008^*^
	Day 10	2.40 ± 0.52	3.10 ± 0.74	2.458^*^	0.024^*^
Metal	Black powder				
	Day 1	2.20 ± 0.63	3.80 ± 0.63	5.657^*^	<0.001^*^
	Day 2	2.0 ± 0.82	3.0 ± 0.67	3.000^*^	0.008^*^
	Day 10	1.50 ± 0.85	1.90 ± 0.74	1.124	0.276
	SPR				
	Day 1	2.60 ± 0.70	3.20 ± 0.63	2.012	0.059
	Day 2	1.70 ± 0.82	2.90 ± 0.74	3.432^*^	0.003^*^
	Day 10	1.80 ± 0.79	2.90 ± 0.88	2.952^*^	0.009^*^
	CA				
	Day 1	4.10 ± 0.74	4.60 ± 0.52	1.756	0.096
	Day 2	4.20 ± 0.63	4.40 ± 0.52	0.775	0.449
	Day 10	1.30 ± 0.48	3.50 ± 0.71	8.124^*^	<0.001^*^
Plastic	Black powder				
	Day 1	1.70 ± 0.82	3.20 ± 0.79	4.160^*^	0.001^*^
	Day 2	1.60 ± 0.84	2.50 ± 0.53	2.862^*^	0.010^*^
	Day 10	1.60 ± 0.84	1.20 ± 0.42	1.342	0.202
	SPR				
	Day 1	2.30 ± 1.16	2.70 ± 0.82	5.657^*^	<0.001^*^
	Day 2	1.20 ± 0.42	2.80 ± 0.79	2.415^*^	0.027^*^
	Day 10	1.40 ± 0.71	2.30 ± 0.95	1.964	0.065
	CA				
	Day 1	3.0 ± 0.82	3.60 ± 0.52	1.964	0.065
	Day 2	2.30 ± 1.42	2.70 ± 0.82	0.771	0.453
	Day 10	2.10 ± 0.74	2.90 ± 0.74	2.424^*^	0.026^*^

t: Student t-test for comparing between the groups

*Statistically significant at *p* ≤ 0.05

## Discussion

Fingerprints are considered to be a key role and the most valued tool in crime scene investigation. The detection of latent fingerprints is practically a challenging analytical problem, where detection of very small quantities of specific chemical compounds is required (Cadd et al. 2015). Consequently, the current study was conducted to evaluate the possibility of recovery of submerged latent fingerprints on non-porous surfaces using different techniques. Black powder, SPR and CA were used in the current study as these methods are the most commonly used techniques and they are fairly adaptable in their applicability (Yamashita and French 2011).

The groomed fingerprints were used in the current study. Although International Fingerprint Research Group **(IFRG)** stated that natural fingerprints is preferable, but groomed fingerprints were accepted in cold weather (18 °C) (Almog et al. 2014).

Home- made cyanoacrylate fuming chamber was used as it’s cheap, easy and could be made from available items. Different agencies and institutions especially in developing countries cannot afford the acquisition of fuming cabinet with programmable humidity. In the current study; all surfaces subjected to cyanoacrylate fuming were placed under the same experimental conditions (temperature, humidity and time).

In order to assess the effect of water salinity (fresh versus sea water) on latent prints development and the effect of various methods on the same substrate, it was better to leave the surface two hours to dry before applying SPR. This is to exclude effect of surface wetness during the comparison of black powder, SPR and CA.

The present study revealed that; successful recovery of good and very good quality of latent fingerprints is possible following submersion in different aquatic environments. In crime scenes, it’s unlikely that fingerprint processing and enhancement takes place immediately after deposition especially in underwater crime scene (Soltyszewski et al. 2007). Therefore, fingerprints were examined at different intervals; 1, 2 and 10 days.

In the present work, either in sea or fresh water, the duration of submersion in water has its effects with a marked diminished quality of fingerprints in longer duration (10 days). However, prints of good visibility (score 4) were still detected at 10th days when CA fuming was used. This could be of practical importance during examination of such evidences whatever the nature of the surfaces.

The reduced quality of developed fingerprints with increasing the time elapsed since deposition may be explained in the light of the fact that; fingerprint composition changes through various chemical, biological and physical processes resulting in the aged composition (Cadd et al. 2015). Initial compounds are lost through various processes including degradation, metabolism, migration, oxidation and polymerization. The longer aging periods may result in greater degradation of fingerprint components (Girod et al. 2012).

Former research exploring these changes has chiefly focused on lipid components; fatty acids, wax, esters, triglycerides, cholesterol and squalene within fingerprints, as these tend to decrease significantly in concentration over time (Mong et al. 1999; Weyermann et al. 2011). Additionally, water, bottom mud, sands and other factors can very easily cause prints to fade faster. Trapecar study (Trapecar 2012a) demonstrated similar results in his study made on wet foil, where he assumed that the quality of the developed fingerprints on objects found in water would depend on the length of submersion.

Similarly, Soltyszewski et al. (Soltyszewski et al. 2007) confirmed the possibility of recovering fingerprints deposited on glass slides submerged in river, sea, tap, or distilled water. However they used; aluminum powder, ferromagnetic powder, and CA. They found a decrease in latent fingerprint visualization with increasing the duration of submersion. In contrast to the results of the present study, they stated that prints submerged for 1 and 7 days were on average of good to very good visibility in all used techniques at 5 °C. This may be referred to the difference in methods of visualization, temperature of water between the two studies and the effect of sand and mud on reduced quality of visualization.

The present study also demonstrated that, the highest percentage of good and very good quality (score 4, 5) of fingerprints was detected when CA technique is used.

Additionally, in comparing the three studied methods, a significant difference (*p* ≤ 0.05) was demonstrated among them in most of the examined prints.

Similar results were obtained in another study by Trapecar (Trapecar 2012b), where the examined glass and metal surfaces were exposed to the influences of stagnant water during different time intervals. He concluded that, the best results were achieved with CA. Although, silver special powder, SPR (black and white) and CA were used for prints development. Moreover, the time intervals were; 4 h, 1, 2 and 7 days.

In contrast, Trapecar (Trapecar 2012a), showed that SPR is the best method for development of fingerprints from wet transparent foil surface submerged in stagnant water during different time intervals. It could be attributed to the different nature of the surface used and to enhancement technique being applied while the surface is still wet, while in the current study various techniques were used after the surfaces being dried.

A study investigated the effect of aquatic environment, as a destructive crime scene condition, on the quality of fingerprints. Water has an effect on the survivability of latent prints, and their successful development (Dhall and Kapoor 2016). Sea water had more destructive effect due to its salinity; this could be explained by the good quality of fingerprints recovered from fresh water versus sea water as revealed in the current study.

## Conclusion

The present study concluded that it is possible to recover latent prints submerged in water on different **non porous dried surfaces** with the best visualization method using CA either in fresh or sea water. Also, the duration of submersion affects the quality of fingerprints developed; the longer the duration, the worse the quality is. In addition, this study has revealed that the exposure to high salinity i.e. sea water has more damaging influence on the quality of detected fingerprints.

This study showed that any piece of evidence recovered from underwater should be tested for prints, no matter the amount of time spent beneath the surface.
